# The Cell Birth Marker BrdU Does Not Affect Recruitment of Subsequent Cell Divisions in the Adult Avian Brain

**DOI:** 10.1155/2015/126078

**Published:** 2015-02-11

**Authors:** Anat Cattan, Amir Ayali, Anat Barnea

**Affiliations:** ^1^Department of Zoology, Tel Aviv University, 6997801 Tel Aviv, Israel; ^2^Sagol School of Neuroscience, Tel Aviv University, 6997801 Tel Aviv, Israel; ^3^Department of Natural and Life Sciences, The Open University of Israel, 108 Ravotsky Street, P.O. Box 808, 43107 Ra'anana, Israel

## Abstract

BrdU is commonly used to quantify neurogenesis but also causes mutation and has mitogenic, transcriptional, and translational effects. In mammalian studies, attention had been given to its dosage, but in birds such examination was not conducted. Our previous study suggested that BrdU might affect subsequent cell divisions and neuronal recruitment in the brain. Furthermore, this effect seemed to increase with time from treatment. Accordingly, we examined whether BrdU might alter neurogenesis in the adult avian brain. We compared recruitment of [^3^H]-thymidine^+^ neurons in brains of zebra finches (*Taeniopygia guttata*) when no BrdU was involved and when BrdU was given 1 or 3 months prior to [^3^H]-thymidine. In nidopallium caudale, HVC, and hippocampus, no differences were found between groups in densities and percentages of [^3^H]-thymidine^+^ neurons. The number of silver grains per [^3^H]-thymidine^+^ neuronal nucleus and their distribution were similar across groups. Additionally, time did not affect the results. The results indicate that the commonly used dosage of BrdU in birds has no long-term effects on subsequent cell divisions and neuronal recruitment. This conclusion is also important in neuronal replacement experiments, where BrdU and another cell birth marker are given, with relatively long intervals between them.

## 1. Introduction

DNA replication of stem cells during the S-phase can be used to birth-date proliferating cells by supplying exogenous markers that are incorporated into the DNA of the cells. The marker, which is retained in the postmitotic cell, enables the subsequent detection of a cohort of dividing cells that were labeled at a known time and therefore provides a tool by which to quantify neurogenesis, the effect of various conditions on it, and the fate of the new neurons at a later date (reviewed in [1]).

The traditional approach to detection of cell proliferation is by incorporation of [^3^H]-thymidine, using autoradiography [2]. This technique has been used for decades to investigate various processes in the CNS (e.g., [3–6]) and has been invaluable for the study of the time of origin of neurons in various species, including birds [1], rodents [7], and nonhuman primates [8, 9]. However, due to the radioactivity of [^3^H]-thymidine and the relatively long period which is required for autoradiography, over the years analogs have been introduced as an alternative, of which the most commonly used to date is 5-bromo-2′-deoxyuridine (BrdU) [10]. Because [^3^H]-thymidine and BrdU label cells for only about 2 h after injection [11] and perhaps for an even shorter time in birds [12], the time of cell division can be determined. Both markers have advantages and drawbacks (reviewed in [1]) and hence are preferable for some applications and limited for others. One advantage of BrdU is that it can be quickly detected by immunocytochemistry. Moreover, it can be used in combination with immunoperoxidase labeling for phenotypic markers, which can then be detected by bright-field, fluorescence, and confocal microscopy to determine colocalization (e.g., [13]).

**Figure 1:**
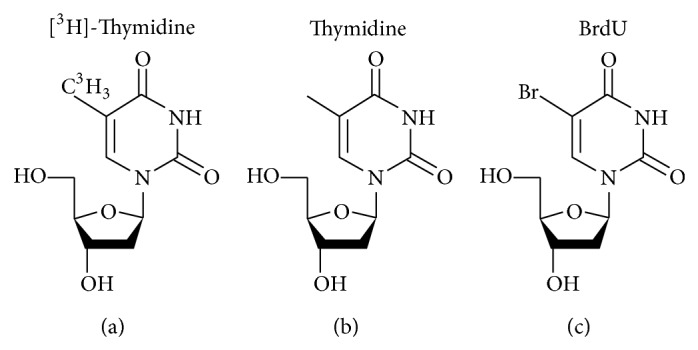
Molecular structures of (a) [^3^H]-thymidine; (b) thymidine; (c) BrdU.

A disadvantage of BrdU is that its molecular structure inherently differs from the natural structure of thymidine, causing steric hindrance (Figure 1). Consequently, BrdU is a toxic mutagenic substance and can cause abnormalities in DNA transcription and protein translation, which could lead to mutation and cell toxicity, compromising the overall health and behavior of the organisms, both in fetal and in postdevelopmental stages [14–16]. In addition, BrdU triggers cell death and the formation of teratomas, alters DNA stability, lengthens cell cycle, and has mitogenic, transcriptional, and translational effects on the cells that incorporate it (reviewed in [17]). The delaying effect of BrdU on DNA synthesis and cell division is probably caused by structural changes in the DNA helixes, which occur when a large amount of BrdU is incorporated into both strands of the DNA [18]. Furthermore, BrdU has been found to cause abnormal proliferation* in vivo* [19], although others found no cytotoxic effects on adult neurogenesis [20].* In vitro*, it has been shown to be toxic to neuronal precursors at the concentrations used in most labeling studies [21].

Since the evidence indicates that BrdU might affect the studied cell population, when its use as a birth-date marker was established, much attention was given to its dosage and the frequency of exposure, in order to minimize toxicity and avoid misleading results (e.g., [10, 11, 22–27]). In adult mammals, a dose of 50 mg/kg given daily for up to 12 days and doses of up to 300 mg/kg did not cause adverse physiological effects or toxic effects in dividing cells in the dentate gyrus [11, 28]. However, it is possible that BrdU is less toxic in adults than in fetuses or juveniles. Whereas all these studies were performed on mammals, in birds such a thorough examination has not been conducted. Instead, BrdU dosage in avian research is similar to that used in fetal mammals, because of the similar high metabolic rates of the two groups. Thus, in birds, commonly used doses are ~60–100 mg/kg body mass (e.g., [29–31]).

In one of our previous studies, we encountered surprising results in a study with zebra finches (*Taeniopygia guttata*). To provide evidence for neuronal replacement, we used two cell birth markers in the same birds: BrdU, to label neurons born 1 or 3 months prior to social manipulation [32], and [^3^H]-thymidine, to label neurons born during the 5 days immediately before the manipulation (unpublished data). We hypothesized that if the total number of neurons remains constant, then as the BrdU^+^ neurons decreased, the [^3^H]-thymidine^+^ ones would increase. Since in previous studies with the same setup [33, 34] we had already labeled neurons that had been born immediately prior to social manipulation with [^3^H]-thymidine, we expected similar patterns of [^3^H]-thymidine^+^ neurons to appear also in the recent study, but this was not the case. Previously, more neurons had been recruited if birds were placed in a complex social setting than if they were kept in a simple one. However, this time no significant differences were found between experimental groups, and recruitment levels were lower and similar to those previously observed in birds that were kept in a simple social setting. Moreover, in our recent study, time was a factor: in the brain region HVC, the longer interval between BrdU and [^3^H]-thymidine treatments (3 months) had a significantly greater negative effect on recruitment of [^3^H]-thymidine^+^ neurons, compared with that when the interval between treatments was shorter (1 month).

These unexpected findings raised the possibility that BrdU might have interfered with the mechanism of neurogenesis, as follows: BrdU, the first marker used, incorporated into dividing VZ mother cells; these gave rise to BrdU^+^ neurons which migrated to their final destination in the brain, where we recorded them. However, BrdU incorporation into the mother cells might have altered them and, by doing so, negatively affected their ability to divide again and produce more neurons. If so, then this effect was then reflected in the unexpected results that we obtained when we later treated the same birds with the second birth-date marker, [^3^H]-thymidine, and recorded [^3^H]-thymidine^+^ neurons. In other words, we allowed for the possibility that BrdU incorporation into dividing VZ stem cells might block or change their ability to divide again and give rise to more daughter cells. As a result, fewer mother cells would produce new neurons in the future and this would affect the number of neurons available for recruitment in the brain. Under such a scenario, the experimental tool that we applied may have interfered with the mechanism that it was intended to investigate.

Accordingly, the aim of this study was to address the possibility, which has not been tested before, that BrdU, used as a research tool, might alter neurogenesis in the adult avian brain. To test this we compared the results of [^3^H]-thymidine labeling when no BrdU was involved (control) and when BrdU was given 1 or 3 months prior to [^3^H]-thymidine treatment. If BrdU interferes with subsequent divisions of the same cells, then the control will yield more [^3^H]-thymidine^+^ neurons than in the other groups. Moreover, if time negatively affects the process, then a 1-month interval between treatments will yield more [^3^H]-thymidine^+^ neurons than a 3-month interval.

Several studies already used two birth-date markers in the same animal, in order to label neuronal subpopulations of different ages and to reveal the relationships between these subpopulations in response to specific conditions (reviewed by Llorens-Martín and Trejo [35]). All these studies were done in mammals, and many of them used other thymidine analogs, IdU and CldU, with relatively short intervals between them (hours to a few weeks). However, to the best of our knowledge our study is the first to test whether the marker which is used first might interfere with subsequent cell divisions and affect the outcome of a second marker, which is used in the same animal after relatively long intervals. More specifically, we tested the validity of using BrdU as the first marker, in light of the evidence that indicates its possible toxicity (see above). Other studies have used BrdU as a single marker or combined it with another one, simultaneously or at short intervals (e.g., [10, 11]). Our experimental design was thus intended to indicate whether BrdU has a long-term effect on the future functionality of neurogenesis. This can be highly relevant to experiments which examine the dynamics of neuronal replacement by using two birth markers.

## 2. Materials and Methods

### 2.1. Experimental Design

**Figure 2:**
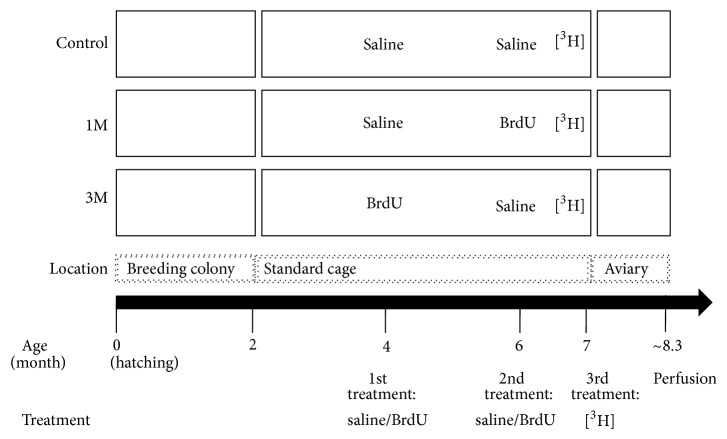
A schematic illustration of the experimental design and groups. See text for details.

As noted in the Introduction, the present study derives from a previous one from our laboratory [32] and, therefore, the experimental design and methods are similar to those described there. A schematic presentation of the experimental design and groups is presented in Figure 2. The study was approved by the Tel Aviv University Institutional Animal Care and Use Committee (permit # L-09-003) and was carried out in accordance with its regulations and guidelines regarding the care and use of animals for experimental procedures. Food and water were provided* ad libitum* and consisted of millet, flies maggots, green vegetables, and crushed hard boiled eggs.

Male zebra finches (*Taeniopygia guttata*) were reared in outdoor breeding colonies at the I. Meier Segals Garden for Zoological Research at Tel Aviv University and banded with numbered plastic rings for individual identification. At the age of two months, when experimental males (ExMs) become independent, they were removed from their native colony, randomly allocated to an experimental group, and housed outdoors in a standard cage (68 × 34.5 × 45.5 cm) with three other unrelated adult individuals (one female and two males). This was done to avoid the stress that, in this very social species, might result from isolation. Each cage was visually isolated from the outside environment and placed far enough from other cages to achieve auditory isolation.

The ExMs remained in this setting until they were about four months old. According to their designated experimental group, each ExM then received the first treatment, of five daily intramuscular injections of either 130 *μ*L saline (NaCl 0.9%, as a sham treatment) or 130 *μ*L (i.e. 100 mg/kg) of the cellular birth-date marker 5-bromo-2′-deoxyuridine (BrdU; SigmaUltra, diluted 10 mg/mL in sterile water; Sigma). This BrdU dose is similar to that used by others to study adult neurogenesis in birds (e.g., [29–32]). The ExMs were kept under the above conditions for two more months, when they received a second treatment of five daily intramuscular injections of either 130 *μ*L saline or 130 *μ*L BrdU, according to their experimental group. After an additional month, all ExMs received a third treatment of five daily intramuscular injections of 50 *μ*L of a radioactive form of thymidine (PerkinElmer NET-027, [^3^H]-thymidine, 5 mCi), which is a marker of DNA synthesis and therefore of cell birth. Two hours after the last [^3^H]-thymidine injection, each ExM was moved to a large outdoor aviary (1.5 × 1.5 × 2 m) where it encountered a complex social environment with a preexisting group of 40–45 adults of both sexes, all of them strangers to the ExM. Birds in any given aviary could neither hear nor see those in the other aviaries. The ExMs remained in the new social environments for 40 d, until they were sacrificed.

All cages and aviaries were exposed to natural conditions (10.1–14.7 h light per day and mean 12–30°C daily temperature). Our birds are able to breed any time of the year under these conditions. Therefore and because the ExMs for each experimental group were obtained at all times of the year, seasonal changes in temperature and photoperiod were unlikely to have affected the outcome of our study.

To recapitulate, in the control group (*n* = 7), ExMs received saline injections in the first and second treatments, followed by [^3^H]-thymidine injections in the third treatment, and were then transferred to a new social setting. Hence, in this group there was no BrdU treatment. In group 1 M (1 month; *n* = 7), ExMs received saline injections in the first treatment and BrdU injections in the second treatment, followed by [^3^H]-thymidine injections in the third treatment, and then were transferred to a new social setting. Hence, in this group there was a one-month interval between BrdU and [^3^H]-thymidine treatments. In group 3 M (3 months; *n* = 6) ExMs received BrdU injections in the first treatment and saline injections in the second treatment, followed by [^3^H]-thymidine injections in the third treatment, and were transferred to a new social setting. Hence, in this group there was a three-month interval between BrdU and [^3^H]-thymidine treatments.

ExMs were sacrificed 40 d after being introduced into their new social setting. Previous work with another songbird, the canary (*Serinus canaria*), showed that neurons born in adulthood can take from 8 d [36] to 30–40 d [37] to reach their final destination in the telencephalon. Thus, in all groups we probably recorded labeled neurons that had already reached their final destination. The common denominator among all groups is that [^3^H]-thymidine was given prior to the social change, while BrdU was given either one or three months before [^3^H]-thymidine and social change. Thus, the results can show whether BrdU affects subsequent neurogenesis (reflected by the number of [^3^H]-thymidine^+^ neurons in the various groups) and if so whether time plays a role in this process.

### 2.2. Body and Brain Mass

Body mass was used as indirect evidence for overall health and recorded four times: on the first day of each of the three treatments and at the end of the experiment. At the end of the experiment, following perfusion (see below), brains were weighed.

### 2.3. Histology, Immunohistochemistry, and Autoradiography

Forty days after the last [^3^H]-thymidine injection, ExMs were weighed, sacrificed with an overdose of anesthesia (0.06 mL of Ketalar diluted 10 times followed by 0.06 mL of xylazine), and fixed with an intracardiac perfusion with saline followed by 4% paraformaldehyde in 0.1 M phosphate buffer (PB; pH 7.4). Brains were removed, weighed, and transferred into PB at 4°C. After 1-2 d, brains were dehydrated in alcohols, embedded in polyethylene glycol, blocked, and cut transversely at 6 *μ*m intervals.

**Figure 3:**
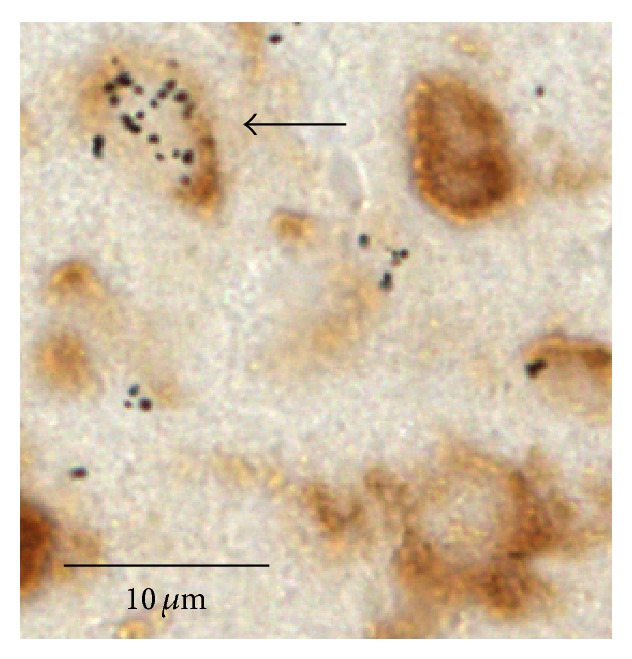
A new neuron (marked with an arrow) labeled with [^3^H]-thymidine (expressed by the silver grains) and stained with anti-Hu (brown).

**Figure 4:**
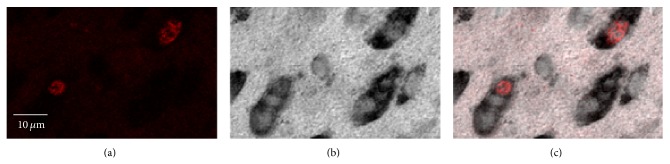
Three confocal images of the same brain tissue demonstrating double-labeling of Hu (brown) and BrdU (fluorescence). Two (red nuclei) of the several neurons (Hu^+^, brown) shown in this field were born at the time of BrdU injections. (a) BrdU labeling. (b) Hu labeling. (c) Colocalization of BrdU and Hu labeling.

Two sets of serial sections (at intervals of 120 *μ*m) were mounted on slides (Superfrost PLUS), using a solution of 0.1% BSA (albumin bovine, minimum 98%) in PBS. Set A was stained to enable [^3^H]-thymidine^+^ neurons identification. Since our main goal was to determine whether an earlier exposure to BrdU interferes with future cell divisions, set A was used to obtain the data to answer this question. We followed a previous protocol from our laboratory (for details see [32]). In brief, sections were incubated with anti-HuC/HuD mouse IgG_2b_, a primary antibody that is affixed specifically to neurons [38], and then with DAB which stained the neurons in the tissue brown. Sections then went through autoradiography and were developed after 4 weeks. This procedure yielded neurons that were stained brown and new [^3^H]-thymidine^+^ neurons that were brown with silver grains (Figure 3). Set B was stained for the identification of BrdU^+^ neurons and was only used to validate the BrdU treatment: namely, to ensure that BrdU had been incorporated into the dividing cells prior to the thymidine treatment. For this set we used an immunohistochemical procedure that had been previously applied in our laboratory (for details see [32]) and is similar to that which had been used by others [30]. This procedure yielded neurons that were stained brown and new BrdU^+^ neurons that were brown with fluorescent red nuclei (Figure 4).

### 2.4. Mapping and Quantification

**Figure 5:**
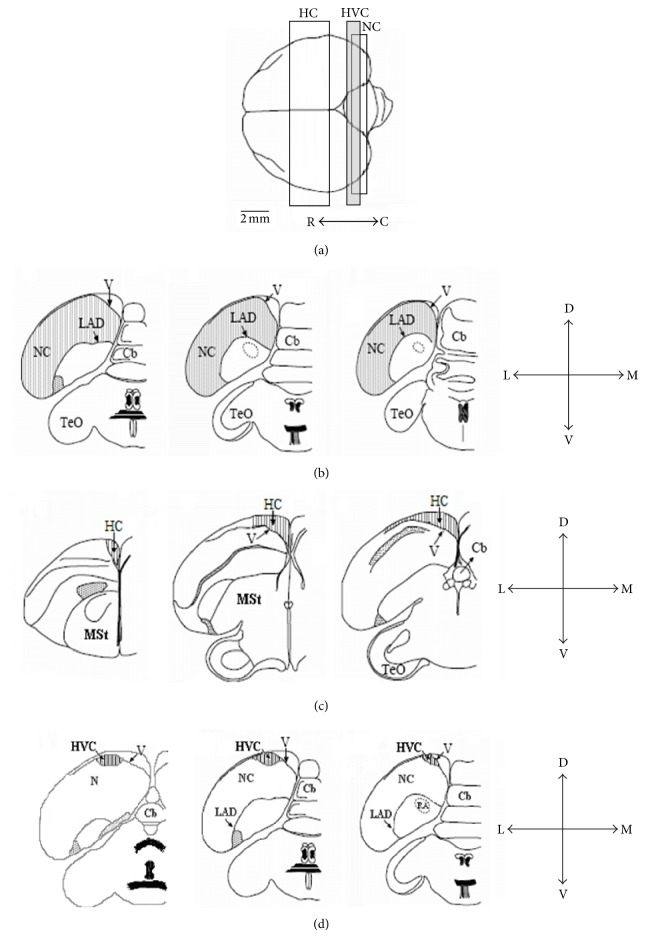
Schematic views of the three investigated brain regions. (a) Top view of the brain: rostral section is to the left and caudal section is to the right. We indicate the range within which frontal sections were taken from the nidopallium caudale (NC), hippocampus (HC), and HVC. Several sections were sampled along the rostrocaudal axis of each brain region (for details, see text); only three sections are shown here: the most rostral, the middle, and the most caudal (from left to right) in NC (b), HC (c), and HVC (d). Cerebellum: Cb, lamina arcopallialis dorsalis: LAD, lateral ventricle: V, nidopallium: N, nucleus robustus arcopallii: RA, and tectum opticum: TeO. Orientations: dorsal: D, lateral: L, ventral: V, and medial: M, adapted from [43] and illustrated by A. Cattan.

We counted new [^3^H]-thymidine^+^ neurons in three brain regions: nidopallium caudale (NC), HVC, and hippocampus (HC) (Figure 5(a)). The NC was chosen because it includes auditory relays that probably play a role in vocal communication and in the processing of other types of auditory inputs [39, 40]. The HVC is part of the song control system and is also involved in vocal communication (reviewed by [41]). HC is known to be involved in the processing of spatial information [42]. These brain regions were previously investigated in our laboratory and neuronal recruitment in them was affected by social manipulation [32, 34, 43]. In addition, these brain regions have boundaries that are easy to recognize in transverse sections: for the NC and HVC we followed the criteria described by [33] and for the HC those described by [34].

The NC stretches rostrocaudally over a distance of 1200 *μ*m, along which we sampled five sections. The middle section went through the robust nucleus of arcopallium (RA) at its largest diameter (P1.2 in the canary atlas [44]). Two more sections were obtained, rostrally and caudally, respectively, at distances of 360 and 600 *μ*m from the middle one (Figure 5(b)). In the HC, which stretches over 2000 *μ*m in the rostrocaudal axis, we sampled five sections per brain. The most rostral section corresponded to A4.0 in the canary atlas [44] and the fifth and most caudal one corresponded to A0.2 there. The three other in-between sections were evenly distributed so that, on average, the distance between each mapped section and the next one was 480 *μ*m (Figure 5(c)). In the HVC, which is 240–720 *μ*m along its rostrocaudal axis, we sampled all mounted sections in which it appeared (five to seven per brain). Rostrally, the first section corresponded to AP0.0 in the canary atlas [44] (Figure 5(d)).

We used a computerized brain-mapping system (Neurolucida; Stereo Investigator; Micro-BrightField) to draw the boundaries of the NC, HVC, and HC in each section sampled, enter the position of [^3^H]-thymidine^+^ neurons, count total neuronal densities, and quantify other neuronal parameters (see below). All mapping was done “blind” as to the experimental conditions and by using a 63x objective. Previous work in our laboratory [32] had revealed no hemispheric differences in all regions in the number of labeled neurons per mm^3^. Therefore we mapped sections only from the right hemisphere.

As the NC is a large brain region, after conducting a preliminary evaluation we scanned about 35% of the whole sections, by using the fractionator probe in our mapping system. However, in the HVC and HC, which are relatively small, we completely scanned all the mapped sections, using the meander scan probe. In the HVC and HC we also measured the neuronal nuclear diameters of all of the new neurons. Since the NC is a large region with hundreds of new neurons per section, we measured neuronal nuclear diameters only in the fifth section, in which we sampled six nonoverlapping squares (each of an area of 19,600 *μ*m^2^) randomly chosen by the software and which yielded at least ten [^3^H]-thymidine^+^ neurons.

From the knowledge of section thickness and mean neuronal nuclear diameter of labeled neurons in a certain brain region, we used the Abercrombie stereological correction [45] to estimate the number of [^3^H]-thymidine^+^ neurons per mm^3^ in each brain region. In the NC and HC these estimates were obtained for each section. In the HVC, data from all sections mapped for each brain were pooled, with each brain represented by a single estimate of the number of [^3^H]-thymidine^+^ neurons per mm^3^. This was done because of the variability in the number of sections of this region in each brain, which prevented our comparison of them.

In each brain region we also estimated the density of total neurons ([^3^H]-thymidine^+^ and unlabeled ones) per mm^3^. This was done in the fifth section, in which all neurons were counted in six squares as described above for the neuronal nuclear diameter of new neurons. In the first and last squares we also measured nuclear diameters of all neurons ([^3^H]-thymidine^+^ and unlabeled ones). As explained above, this allowed us to estimate the total number of neurons per mm^3^ in each brain region and also to calculate the percentage of new neurons per mm^3^, out of the total number of neurons.

### 2.5. Statistical Analysis

Most of the statistical analysis was performed using STATISTICA © 8 (StatSoft, Inc.). Analyzing the interaction between sections in the parameter of number of [^3^H]-thymidine^+^ neurons per mm^3^ in HC was performed using SPSS v15, with ANOVA repeated-measures within-subjects contrasts. All data expressed as number of neurons per mm^3^ or proportions were transformed before the statistical analysis using the square root transformation. (These kinds of data, number of discrete elements per unit, tend to have a poison distribution, and the suitable transformation for such a case is the square root transformation [46].) *P* < 0.05 was considered significant throughout. To compare different parameters obtained from several section levels, analysis of variance was performed using Two-Way ANOVA (repeated-measures). Since we had only one uniform discrete categorical variable (number of months between exposure to BrdU and [^3^H]-thymidine), all ANOVA tests used the fixed-effects model (model 1). To analyze the differences in body mass between experimental groups we used Two-Way ANOVA (repeated-measures) in model 1. To analyze the differences in brain mass we used One-Way ANOVA in model 1 [46].

## 3. Results

The main aim of this study was to determine whether treatment with the cell birth marker BrdU affects subsequent cell divisions and the recruitment of neurons that differentiate from these divisions into various brain regions. We thus injected birds with BrdU and either one or three months later treated them with another cell birth marker, [^3^H]-thymidine (groups 1 M and 3 M, resp.). A control group (group C) received only [^3^H]-thymidine treatment. To answer our question, we then compared the number of [^3^H]-thymidine^+^ neurons in the tested brain regions between the three experimental groups. Our prediction was that if BrdU affects subsequent cell divisions and the recruitment of neurons that differentiate from these divisions, then fewer [^3^H]-thymidine^+^ neurons will be found in the two groups that were treated with BrdU (1 M and 3 M), compared to the control. In addition, if the effect of BrdU is time-dependent, we expected fewer [^3^H]-thymidine^+^ neurons in group 1 M, compared to group 3 M.

### 3.1. Densities of [^3^H]-Thymidine^+^ Neurons

**Figure 6:**
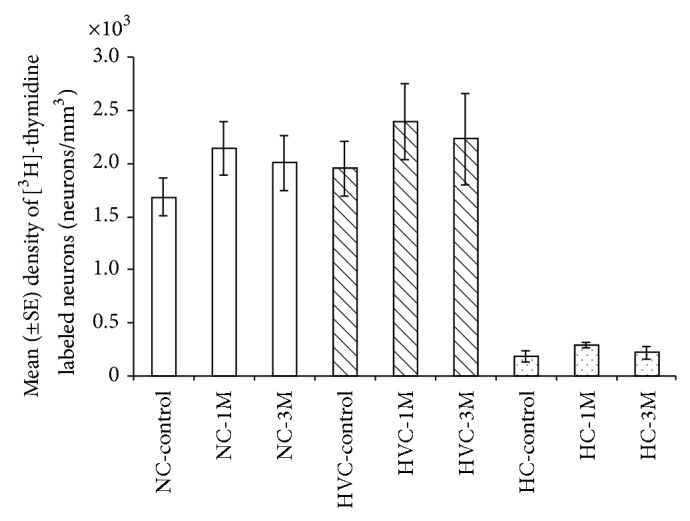
Mean (±SE) densities (neurons/mm^3^) of [^3^H]-thymidine^+^ neurons in NC, HVC, and HC, in the three experimental groups (control; 1 M: one month after BrdU treatment; 3 M: three months after BrdU treatment). In all the three investigated brain regions, no significant differences were found between experimental groups. Sample sizes: control and 1 M = 7 brains; 3 M = 6 brains.

In the three investigated brain regions, we found no significant differences between the experimental groups in the density of [^3^H]-thymidine^+^ neurons (Figure 6; NC: F_(2,17)_ = 1.0508, *P* = 0.371; HVC: F_(2,17)_ = 0.3399, *P* = 0.716; HC: F_(2,17)_ = 1.1787, *P* = 0.331). This indicates that BrdU does not affect subsequent cell divisions and the recruitment of neurons that differentiate from these divisions. This holds true both for the subsequent cell divisions which occur relatively soon (1 month) after BrdU administration and for divisions which occur after longer intervals (3 months).

In addition, in the NC, no significant differences were found between sections (F_(4,68)_ = 1.6420, *P* = 0.173) and no significant interaction was found between sections and groups (F_(8,68)_ = 0.7481, *P* = 0.649); and, accordingly, the overall mean density in this region was 1940 ± 132 [^3^H]-thymidine^+^ neurons/mm^3^. In the HVC, no significant differences were found between groups (F_(2,17)_ = 0.3399, *P* = 0.716); and, accordingly, the overall mean density was 2187 ± 194 [^3^H]-thymidine^+^ neurons/mm^3^. In the HC, significant differences were found between sections (F_(4,68)_ = 7.5078, *P* < 0.001), but no interaction was found between sections and experimental groups (F_(8,68)_ = 0.6892, *P* = 0.699); and, accordingly, the overall mean density in this region was 232 ± 26 [^3^H]-thymidine^+^ neurons/mm^3^.

### 3.2. Percentages of [^3^H]-Thymidine^+^ Neurons

**Figure 7:**
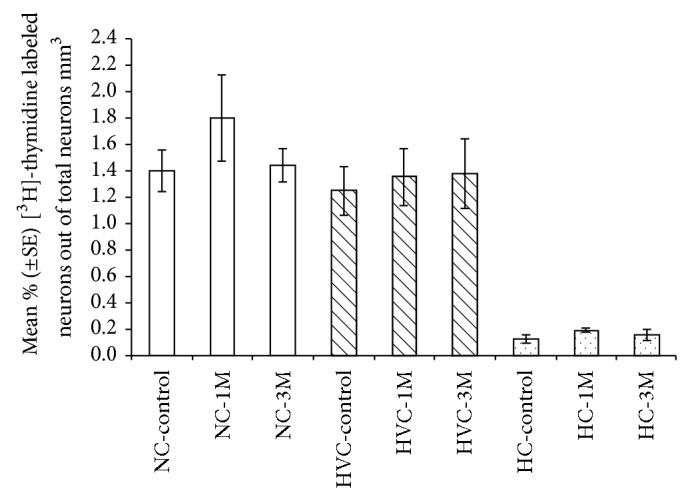
Mean (±SE) percentages (%) of [^3^H]-thymidine^+^ neurons in NC, HVC, and HC, in the three experimental groups (control; 1 M: one month after BrdU treatment; 3 M: three months after BrdU treatment). In all the three investigated brain regions, no significant differences were found between experimental groups. Sample sizes: control and 1 M = 7 brains; 3 M = 6 brains.

A similar outcome, of no significant differences between experimental groups, in the three investigated brain regions, was also obtained when we calculated percentages of [^3^H]-thymidine^+^ neurons (Figure 7; NC: F_(2,17)_ = 0.8405, *P* = 0.448; HVC: F_(2,17)_ = 0.0692, *P* = 0.933; HC: F_(2,17)_ = 0.9245, *P* = 0.415). This indicates again that BrdU does not affect subsequent cell divisions and the recruitment of neurons that differentiate from these divisions, both for divisions which occur relatively soon (1 month) after BrdU administration and for divisions which occur after a longer interval (3 months).

In addition, in the NC, no significant differences were found between sections (F_(4,68)_ = 1.5705, *P* = 0.192) and no significant interaction was found between sections and groups (F_(8,68)_ = 0.7614,  *P* = 0.637); accordingly, the overall mean percentage of [^3^H]-thymidine^+^ neurons in this region was 1.6 ± 0.6%. In the HVC the overall mean percentage of [^3^H]-thymidine^+^ neurons was 1.3 ± 0.1%. In the HC, a significant difference was found between sections (F_(4,68)_ = 7.7432, *P* < 0.001), but since no interaction was found between sections and groups (F_(8,68)_ = 0.6902, *P* = 0.698), we pooled the results from all sections in each brain, and the overall mean percentage of [^3^H]-thymidine^+^ neurons was 0.2 ± 0.02%.

### 3.3. Nuclear Diameter of [^3^H]-Thymidine^+^ Neurons

No significant differences between groups were found in mean nuclear diameter of [^3^H]-thymidine^+^ neurons in either the NC (F_(2,17)_ = 0.1193, *P* = 0.888) or the HC (F_(2,15)_ = 0.6914, *P* = 0.516). Accordingly, overall mean nuclear diameter of NC neurons was 14.7 ± 0.3 *μ*m (*n* = 283 neurons out of 20 brains) and of HC neurons 14.6 ± 0.2 *μ*m (*n* = 241 neurons out of 20 brains). This finding further supports the indication that BrdU does not affect subsequent cell divisions. However, in the HVC significant differences (F_(2,17)_ = 4.5969, *P* = 0.025) were observed between groups in the mean nuclear diameter of [^3^H]-thymidine^+^ neurons. In the control group HVC neurons were significantly bigger than those in groups 1 M (df = 17, *P* = 0.043) and 3 M (df = 17, *P* = 0.049). Accordingly, mean [^3^H]-thymidine^+^ neuronal nuclear diameter in the control group was 14.9 ± 0.4 *μ*m (*n* = 333 neurons out of 7 brains); in group 1 M 13.4 ± 0.3 *μ*m (*n* = 392 neurons out of 7 brains); and in group 3 M 13.4 ± 0.6 *μ*m (*n* = 364 neurons out of 6 brains).

### 3.4. Number of Exposed Silver Grains per [^3^H]-Thymidine^+^ Neuronal Nucleus and Their Distribution

**Figure 8:**
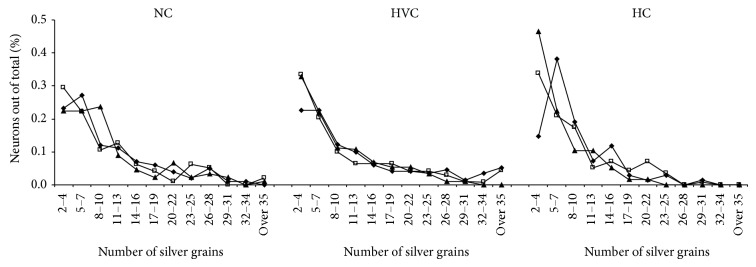
Distributions of the number of exposed silver grains in [^3^H]-thymidine^+^ neurons in NC, HVC, and HC, in brains of birds from the control group (♦; *n* = 7), 1 M (one month after BrdU treatment; □; *n* = 7), and 3 M (three months after BrdU treatment; ▲;  *n* = 6).

In all three investigated brain regions, no significant differences were found between groups in the mean number of silver grains per [^3^H]-thymidine^+^ neuronal nucleus (NC: F_(2,17)_ = 0.0363, *P* = 0.964; HVC: F_(2,17)_ = 0.7189, *P* = 0.501; HC: F_(2,17)_ = 1.0407, *P* = 0.374). This indicates that in each brain region the [^3^H]-thymidine^+^ neurons derive from the same original population and undergo the same number of divisions. Distributions of the number of exposed silver grains in [^3^H]-thymidine^+^ neuronal nuclei further support this conclusion (Figure 8).

### 3.5. Brain and Body Mass

No significant differences were found in body mass between experimental groups (F_(2,18)_ = 1.3651, *P* = 0.280), as well as between experimental stages (F_(3,54)_ = 2.7264, *P* = 0.052), with no interaction between groups and stages (F_(6,54)_ = 0.8207, *P* = 0.558). Accordingly, mean body mass was 13.4 ± 0.8 g (*n* = 27). Similarly, no significant differences were found in brain mass between experimental groups (F_(2,18)_ = 0.0701, *P* = 0.932), and mean brain mass was 0.47 ± 0.02 g (*n* = 21).

## 4. Discussion

Our data indicate that BrdU used as a research tool in the adult avian brain does not affect the recruitment of neurons born after its use, in subsequent cell divisions in the VZ. While we are very much aware of the caution one should take when accepting a null hypothesis based on nonsignificant results, we feel that in this case our conclusion is supported by several semioverlapped as well as independent results. Firstly, no significant differences were found between the control and experimental groups in levels of neuronal recruitment in the three investigated brain regions (NC, HVC, and HC) tested independently. This holds true both for densities of [^3^H]-thymidine^+^ neurons per mm^3^ and for percentages of [^3^H]-thymidine^+^ neurons out of the total neuronal populations. Moreover, time does not affect this outcome, as evident from the similar results obtained when we recorded recruitment of neurons that originated from subsequent cell divisions relatively soon after BrdU administration (1 month) and from divisions that occurred after a longer interval (3 months).

The conclusion that BrdU treatment in the avian brain does not affect subsequent cell divisions in the VZ and the neurons that differentiate from these divisions draws further support from the finding that in most cases there were no group differences in the mean nuclear diameter of [^3^H]-thymidine^+^ neurons (apart from HVC, in which nuclear diameters were larger in the experimental groups, compared to the control). Neuronal nuclear diameter decreases with age [36, 47] and therefore can be used as an indicator for neuronal age. Since mean neuronal nuclear diameters were similar across most groups, we can assume that neuronal age was also similar and, hence, that the [^3^H]-thymidine^+^ neurons were born within the 5 days of treatment.

Two additional findings further strengthen the conclusion that the [^3^H]-thymidine^+^ neurons were derived from the same original population and underwent the same number of divisions: the number of exposed silver grains per [^3^H]-thymidine^+^ neuronal nucleus and their distributions were also similar between groups, in all three investigated brain regions. This supports our conclusion, as the number of exposed silver grains positively correlates to the amount of [^3^H]-thymidine incorporated into the nuclei during the S-phase, when DNA synthesis occurs. If one assumes that all stem cells have a similar duration of S-phase, then the initial uptake of [^3^H]-thymidine^+^ was presumably comparable across groups, since it occurred when all birds were kept under the same conditions. Under such situation, daughter cells that originate from the mitotic event that occurred at the time of treatment are expected to reflect a similar labeling. However, if successive rounds of stem cell divisions take place after the initial labeling, then a dilution of labeling occurs each time, yielding new generations of neurons with less labeling, that is, fewer exposed silver grains per labeled neuron [12, 48]. Hence, the number of exposed silver grains and their distribution in [^3^H]-thymidine^+^ neurons enable us to determine whether these neurons derive from the same population or are the result of several mitotic events [49]. Since the number of exposed silver grains and their distribution were similar across groups in all the investigated brain regions, we conclude that the [^3^H]-thymidine^+^ neurons were a result of the same mitotic events and, hence, that BrdU did not affect it. Finally, it is worth noting that body and brain mass did not differ significantly between experimental groups, and from this and other observations we infer that all the birds remained in good health.

## 5. Conclusions

We investigated in the adult avian brain the possibility that BrdU incorporation into dividing VZ stem cells might block or change their ability to divide again and give rise to more daughter cells. This is important to know because, if so, then using BrdU as a research tool might interfere with the mechanism that it was intended to investigate. However, our data indicate that this is not the case, since in this study BrdU did not affect the recruitment of neurons that were born after its administration. Examination of the correct BrdU dosage in birds has not been done in the past and, therefore, we believe that our conclusion is relevant to experiments that study neuronal replacement in avian systems, since it confirms that the commonly used dosage is acceptable. Our study and its findings are also novel because, to the best of our knowledge, it is the first to test the validity of using BrdU, combined with another birth-date marker in the same animal, with relatively long intervals between markers. Other studies to date have used only one marker or two but simultaneously or at short intervals (e.g., [10, 11]). Therefore, the possible effect of BrdU on neurogenesis and its future functionality, due to its potential toxicity, could not have been detected before. The present study, which indicates that BrdU does not have a long-term effect on the future functionality of neurogenesis, thus has relevance when planning experiments which use two birth markers, for example, when examining the dynamics of neuronal replacement.
